# Assessing changes in regional cerebral hemodynamics in adults with a high-density full-head coverage time-resolved near-infrared spectroscopy device

**DOI:** 10.1117/1.JBO.29.S3.S33302

**Published:** 2024-05-03

**Authors:** Farah Kamar, Leena N. Shoemaker, Rasa Eskandari, Daniel Milej, Darren Drosdowech, John M. Murkin, Keith St. Lawrence, Jason Chui, Mamadou Diop

**Affiliations:** a Western University, Department of Medical Biophysics, London, Ontario, Canada; b Lawson Health Research Institute, London, Ontario, Canada; c Western University, Department of Orthopaedic Surgery, London, Ontario, Canada; d Western University, Department of Anesthesia and Perioperative Medicine, London, Ontario, Canada

**Keywords:** cerebral oximetry, near-infrared spectroscopy, time-resolved near-infrared spectroscopy, high-density near-infrared spectroscopy, carotid compression, phenylephrine

## Abstract

**Significance:**

Cerebral oximeters have the potential to detect abnormal cerebral blood oxygenation to allow for early intervention. However, current commercial systems have two major limitations: (1) spatial coverage of only the frontal region, assuming that surgery-related hemodynamic effects are global and (2) susceptibility to extracerebral signal contamination inherent to continuous-wave near-infrared spectroscopy (NIRS).

**Aim:**

This work aimed to assess the feasibility of a high-density, time-resolved (tr) NIRS device (Kernel Flow) to monitor regional oxygenation changes across the cerebral cortex during surgery.

**Approach:**

The Flow system was assessed using two protocols. First, digital carotid compression was applied to healthy volunteers to cause a rapid oxygenation decrease across the ipsilateral hemisphere without affecting the contralateral side. Next, the system was used on patients undergoing shoulder surgery to provide continuous monitoring of cerebral oxygenation. In both protocols, the improved depth sensitivity of trNIRS was investigated by applying moment analysis. A dynamic wavelet filtering approach was also developed to remove observed temperature-induced signal drifts.

**Results:**

In the first protocol (28±5 years; five females, five males), hair significantly impacted regional sensitivity; however, the enhanced depth sensitivity of trNIRS was able to separate brain and scalp responses in the frontal region. Regional sensitivity was improved in the clinical study given the age-related reduction in hair density of the patients (65±15 years; 14 females, 13 males). In five patients who received phenylephrine to treat hypotension, different scalp and brain oxygenation responses were apparent, although no regional differences were observed.

**Conclusions:**

The Kernel Flow has promise as an intraoperative neuromonitoring device. Although regional sensitivity was affected by hair color and density, enhanced depth sensitivity of trNIRS was able to resolve differences in scalp and brain oxygenation responses in both protocols.

## 1 Introduction

Surgical procedures requiring general anesthesia can lead to impaired oxygen supply to the brain, increasing the risk of cerebral injury. Cerebral desaturation occurs in up to 25% to 37% of all cardiac patients and 69% to 76% of patients undergoing high-risk procedures, such as carotid endarterectomy.^1^^–^^3^ It is particularly a risk during shoulder surgery due to low blood pressure caused by performing the procedure in a semi-upright position (i.e., beach chair position).^4^ Notably, up to 80% of patients undergoing surgery in the beach chair position will require blood pressure interventions to mitigate the risk of cerebral desaturation.^3^^,^^5^ Interventions include re-positioning patients, administering vasopressors to increase mean arterial blood pressure (MAP),^6^ and transfusion in the case of anemia. Detecting evidence of cerebral desaturation is, therefore, a critical component of clinical management to enable prompt administration of treatments, thereby improving patient outcomes.^6^^,^^7^

Cerebral oximeters are now widely used intraoperatively to assess tissue oxygen saturation (${\mathrm{StO}}_{2}$) and detect desaturation events.^6^^,^^8^ However, commercial cerebral oximeters typically have two channels for bilateral monitoring of the frontal watershed regions.^9^ This limited spatial coverage may result in undetected regional cerebral desaturation events.^10^ A further concern with such systems, which use continuous-wave (cw) near-infrared spectroscopy (NIRS) technology, is their susceptibility to extracerebral signal contamination and limited sensitivity to the adult brain. Advances in NIRS technology are providing promising alternatives that could overcome the abovementioned limitations. For example, Kernel Flow^11^^,^^12^ is a new NIRS device with full-head coverage, high density of optodes, and is based on time-resolved NIRS (trNIRS) technology,^13^ which provides greater sensitivity to the adult brain compared to cwNIRS.^14^^–^^16^ However, the feasibility of using this device for neuromonitoring during surgery is unknown. Therefore, we investigated the possibility of using Kernel flow^11^^,^^12^ to monitor regional cerebral blood oxygenation during surgery.

The feasibility study was conducted using two hemodynamic protocols to (1) investigate the ability of the Flow system to simultaneously measure cerebral oxygenation across multiple cortical regions and (2) compare cerebral hemodynamic responses derived using depth-sensitive trNIRS parameters. The first protocol involved applying digital carotid compression (DCC) to healthy volunteers to assess the device’s ability to detect transient decreases in oxygenation. This simple technique induces a decrease in middle cerebral artery blood flow, similar to an occlusion of the internal carotid artery, and is commonly used to assess collateral blood flow during neurovascular surgeries.^17^ Based on previous studies monitoring ${\mathrm{StO}}_{2}$ during DCC,^18^^,^^19^ it was hypothesized that all probes on the ipsilateral hemisphere would exhibit a decrease in ${\mathrm{StO}}_{2}$.^18^

The second protocol assessed the feasibility of using the device for intraoperative neuromonitoring of patients undergoing elective shoulder surgery. Specifically, the effects of phenylephrine (PE) on cerebral oxygenation were investigated. PE is a vasopressor that has been shown to reduce ${\mathrm{StO}}_{2}$.^20^^,^^21^ Importantly, PE is known to cause systemic vasoconstriction without directly affecting cerebral vasomotor tone.^22^ Therefore, we hypothesize that the effects of PE would be similar across the entire cortex. Additionally, analysis conducted using various depth-sensitive trNIRS parameters was expected to reveal differences in scalp and brain responses to PE administration.

## 2 Methods

### 2.1 Instrumentation

**Fig. 1:**
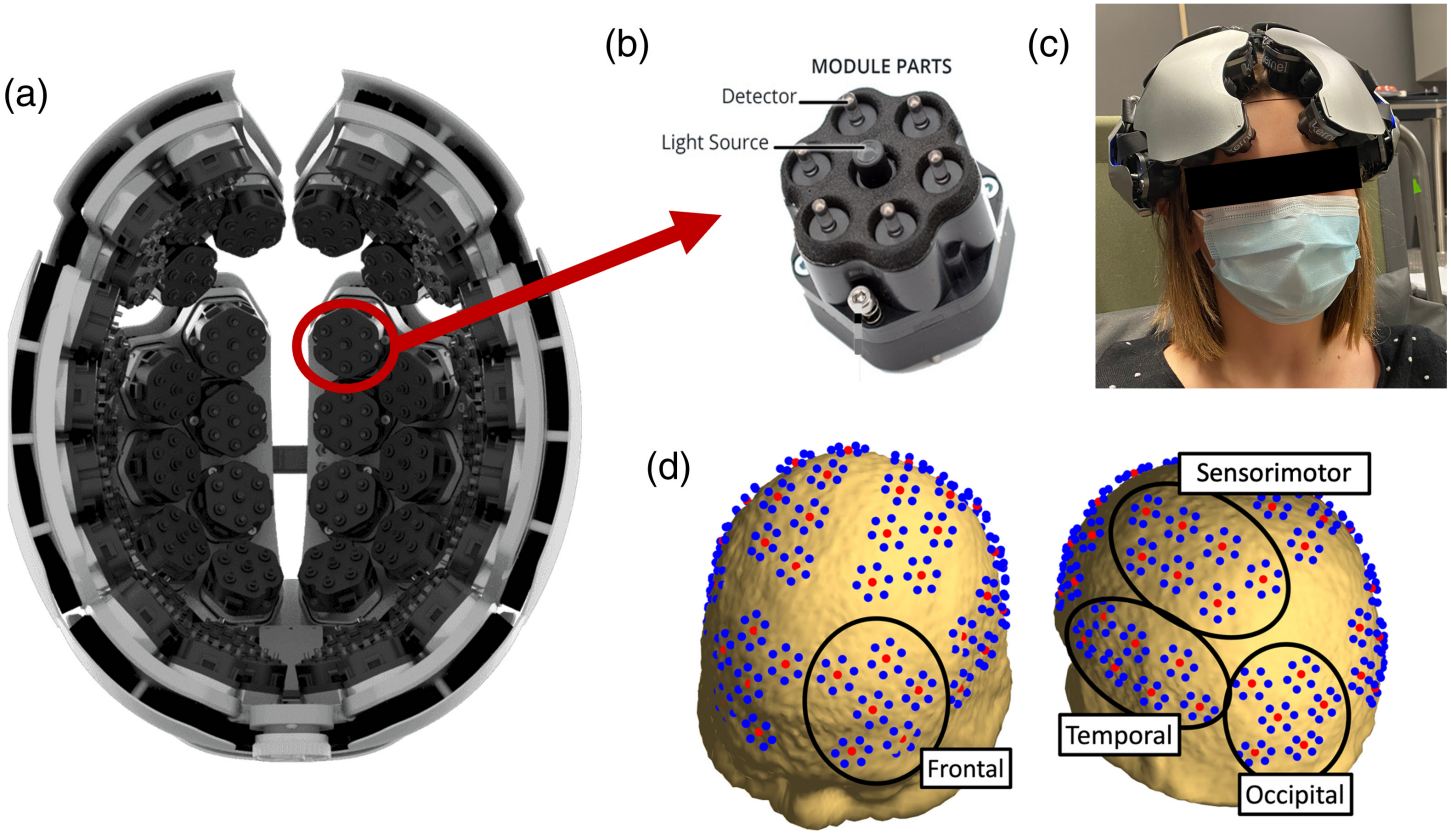
(a) Kernel Flow system showing the inside of the helmet. (b) One of the Kernel Flow modules with the light source in the middle and detectors around the perimeter. (c) Kernel Flow on a participant’s head. (d) Kernel Flow probe placement on a standard head model.^23^ Red dots depict laser sources, and blue dots are detectors. The black circles show the division of the eight brain regions (two hemispheres, each divided into frontal, sensorimotor, temporal, and occipital region). Images in panels (a) and (b) are adapted from Ref. 24.

Kernel Flow^11^^,^^12^ is a trNIRS device that provides high-density, full-head coverage. The system is equipped with 52 hexagonal modules (Fig. 1), each having 2 laser sources in the middle, emitting at 690 and 850 nm, and 6 detectors at the vertices located 10 mm from the sources. There are 52 source locations and 312 detectors. The system provides up to 2206 potential channels with varying source–detector distances (SDDs); 312 are short-distance channels (SDD = 10 mm), and 1894 are long-distance channels (SDD between 20 and 60 mm) across the head. For this study, intramodular channels were classified as short channels (SDD = 10 mm), and cross-modular channels with SDD≥25  mm were classified as long channels. The placement of the optical probes is divided into eight plates that roughly cover the brain’s four lobes and will be referred to as frontal, temporal, occipital, and sensorimotor [Fig. 1(d)]. Additionally, the system uses spring-loaded light pipes for transmission and detection to improve scalp coupling. The device weighs 2.2 kg and has a sampling rate of 7.1 Hz with a built-in temperature sensor and accelerometer.

### 2.2 Experimental Protocols

#### 2.2.1 Protocol 1: digital carotid compression

All procedures were approved by the Health Sciences Research Ethics Board at Western University, which adheres to the guidelines of the Tri-Council Policy Statement for research involving humans. Participants provided written informed consent following written and verbal explanations of the experimental procedures. Ten healthy, young volunteers (Table 1) were recruited to participate in the study. Fitzpatrick Skin Type^25^ and hair color were recorded. The former is based on skin tone and how reactive the skin is to sunlight, ranging from I (light and easily sunburns) to VI (dark and does not typically sunburn). The exclusion criteria included pre-existing neurological, psychiatric, or vascular disease diagnoses. Participants were seated upright with the Kernel Flow on their head and instructed to remain as still as possible during the experiment. The experimental protocol started with a 30-s baseline period, followed by 30-s carotid compression, and ended with a 30-s recovery period. DCC involved applying light pressure to the right common carotid artery at a location ∼1  cm superior to the clavicle.^18^ DCC was repeated twice in each participant, and the trial showing the most pronounced hemodynamic change was used for data analysis.

**Table 1:** Healthy volunteer demographics.

Volunteer ID	Age	Sex	Fitzpatrick skin type	Hair color	Classification	Overall active channels (%)
1	30	M	IV	Balding	Light	39
2	29	F	III	Dark brown	Dark	6
3	23	F	IV	Black	Dark	5
4	38	M	II	Blonde	Light	44
5	22	F	III	Dark brown	Dark	8
6	31	F	II	Dark brown	Dark	6
7	28	M	III	Light brown	Light	16
8	25	M	VI	Black	Dark	9
9	27	F	III	Light brown	Light	10
10	22	M	IV	Black	Dark	9

#### 2.2.2 Protocol 2: Phenylephrine administration during shoulder surgery

**Fig. 2:**
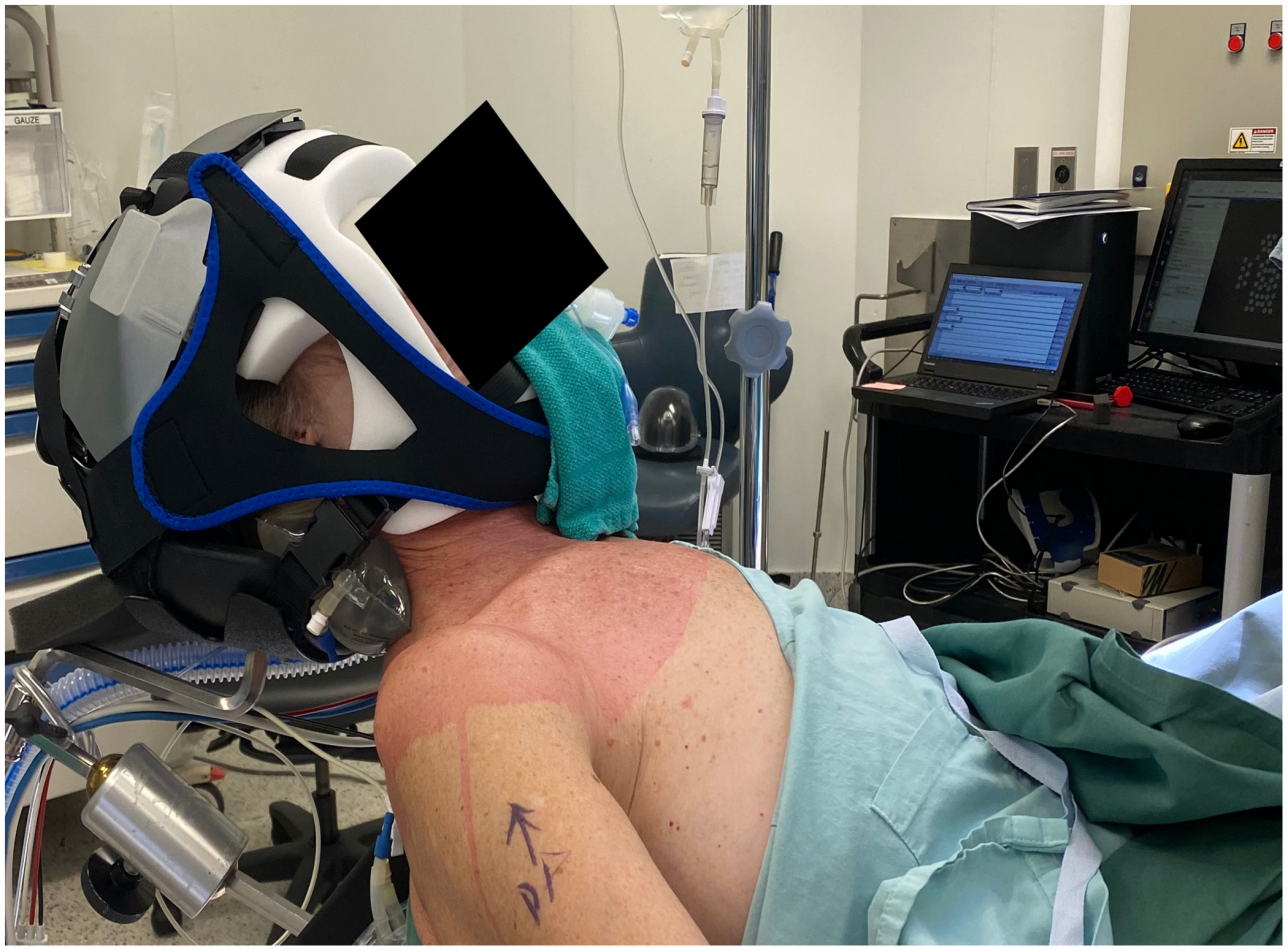
A shoulder surgery patient with the Kernel flow system on the head. The device was stabilized with a head strap.

Adult patients undergoing elective shoulder surgery at St. Joseph’s Hospital (London, Ontario) were prospectively recruited between October 2022 and March 2023. All procedures were approved by the Health Sciences Research Ethics Board at Western University (REB# 115021), which adheres to the guidelines of the Tri-Council Policy Statement for research involving humans. Participants (Table 2) provided written informed consent following written and verbal explanations of the protocol. Once the patient was anesthetized, intubated, and placed in the beach chair position, the Kernel Flow was placed on the head and stabilized with a head strap (Fig. 2). To avoid overheating, a forced-air cooling system (Bair Hugger, 3M Medical) was used to blow cool air at the Flow device. Data were collected continuously throughout the surgical procedure, and instances of PE administration were noted. MAP and heart rate (HR) measurements were acquired using hospital equipment every 5 min. To investigate the effect of PE, 1 min prior to the PE administration was used as the baseline, and 5 min post-injection were included in the analysis.

**Table 2:** Demographics of the patients included in the analysis.

Patient ID	Surgery side	Sex	Age	Weight (kg)	Height (cm)	Shoulder surgery type	Number of PE bolus injections
5	R	F	75	69	160	Arthroplasty reverse	6
7	L	M	69	92	173	Arthroplasty reverse	4
12	R	M	66	92.5	175	Shoulder arthroplasty total	2
13	R	M	68	115	180	Arthroplasty reverse	3
23	R	F	60	67.5	147	Arthroplasty reverse	1

### 2.3 Data Processing

Raw data were uploaded to Kernel’s cloud-based pre-processing pipeline, which starts by removing poor-contact channels based on the shape of the distribution of times-of-flight (DTOF), photon counts, scalp coupling index, and HR peak power.^12^ After preprocessing, we received the pruned data containing only “good” quality channels. The pipeline then detects the peak of each DTOF and defines an integration window for computing the statistical moments: 10% of the peak on the rising edge and 1% on the trailing edge of the histogram. Thereafter, three statistical moments are calculated: (1) the total number of photons (N, i.e., the zeroth moment); (2) the mean time of flight (⟨t⟩, i.e., the first moment); and (3) the variance (V, i.e., the second moment). Note that higher moments are more sensitive to the brain, whereas lower moments are more sensitive to the scalp.^15^^,^^26^ For the remainder of the paper, the total number of photons (N) at a short SDD will be referred to as $N_{\text{short}}$, and the total number of photons at a long SDD labeled $N_{\text{long}}$. The same nomenclature will be applied to the mean time of flight (⟨t⟩) and the variance (V). The least depth-sensitive measurement is $N_{\text{short}}$, which predominantly reflects the scalp response. Likewise, measurements with the greatest depth sensitivity are derived from $V_{\text{long}}$, which mostly reflects the brain response.^16^^,^^27^

Data were further processed using an in-house pipeline developed in MATLAB (Mathworks Inc., United States). Changes in each moment relative to its baseline value were calculated to generate three different time courses for each wavelength. Time courses were then converted into corresponding absorption changes ($\Delta \mu_{a}(\lambda )$) using sensitivity analysis^28^^,^^29^ [Eq. (1)], where ΔS is the signal change for a given moment and ${\mathrm{SF}}_{S}$ represents the corresponding sensitivity factor (which also dependents on the source-detector distance)

$$\Delta \mu_{a}(\lambda )=\frac{\Delta S}{{\mathrm{SF}}_{S}}.$$ (1)

$\Delta \mu_{a}$ was then converted to oxyhemoglobin (HbO) and deoxyhemoglobin (Hb) concentration changes (Δ[HbO] for HbO, and Δ[Hb] for Hb) using Eq. (2), where $\varepsilon_{\mathrm{HbO}}(\lambda )$ and $\varepsilon_{\mathrm{HbO}}(\lambda )$ are the molar extinction coefficients for HbO and Hb, respectively

$$\Delta \mu_{a}(\lambda )=\varepsilon_{\mathrm{HbO}}(\lambda )\times \Delta [\mathrm{HbO}]+\varepsilon_{\mathrm{Hb}}(\lambda )\times \Delta [\mathrm{Hb}].$$ (2)

Baseline values of [HbO] and [Hb] (i.e., ${\left[\mathrm{HbO}\right]}_{b}$ and ${\left[\mathrm{Hb}\right]}_{b}$) from the literature were assumed for all participants (33 and 24  μM,^30^^,^^31^ respectively). Relative concentration changes, calculated from the different moments and SDDs, were used to estimate ${\mathrm{StO}}_{2}$ changes using the following equation:

$$\Delta {\mathrm{StO}}_{2}=(\frac{\left(\Delta [\mathrm{HbO}]+[\mathrm{HbO}{]}_{b}\right)}{\left(\Delta [\mathrm{HbO}]+[\mathrm{HbO}{]}_{b})+(\Delta \mathrm{Hb}+[\mathrm{Hb}{]}_{b}\right)}-\frac{[\mathrm{HbO}{]}_{b}}{[\mathrm{HbO}{]}_{b}+[\mathrm{Hb}{]}_{b}})\times 100\%.$$ (3)

Detection channels were sorted into eight brain regions based on the four brain lobes of each hemisphere, and regional averages were calculated. Time courses were filtered with a zero-phase filter using a window size of approximately 1.5 s.

### 2.4 Correction for Temporal Drift

The time course of Δ[HbO] and Δ[Hb] exhibited a strong dependence on the device’s temperature, which manifested as temperature-dependent signal drifts. This was particularly evident during the longer monitoring periods in the surgery study (protocol 2). To mitigate the confounding effects of the temperature drift, dynamic bandpass wavelet filtering was implemented. The filtering consisted of applying a continuous wavelet transform to decompose the temperature time course of each module to identify the frequency of temperature drift with maximum power across the monitoring period. This frequency was then used as the lower limit of the bandpass filter for the Δ[HbO] and Δ[Hb] time courses. Across all patients, the lowest frequency of the bandpass filter was 0.008 Hz (period of 125 s). The upper limit of the bandpass filter was consistently 3.1 Hz, as limited by the device sampling rate (7.1 Hz). Each channel was filtered with its module-specific, time-varying bandpass filter. This resulted in 52 unique filters used across the head. This approach could not be applied to the DCC time courses (protocol 1) because the measurements were only 90 s; instead, a simple first-order polynomial was used for detrending.

### 2.5 Statistical Analysis

All data were assessed for normality through a visual inspection of a QQ plot as well as the Shapiro–Wilk test. All statistics were conducted in GraphPad Prism version 9 (GraphPad Software Inc., San Diego), and statistical significance was defined as p≤0.05.

#### 2.5.1 Protocol 1: digital carotid compression

A one-way analysis of variance (ANOVA) was used to compare regional differences in device sensitivity (four levels: frontal, sensorimotor, temporal, and occipital in the ipsilateral hemisphere) as measured by the percentage of good-quality, long-distance channels compared to the total number of expected channels (% of active channels). The impact of hair was also assessed by classifying participants as having “light” hair if they were either bald or had blonde or light brown hair and “dark” hair if they had brown or black hair. An ordinary two-way ANOVA was used to evaluate “light” hair versus “dark” hair (two levels) and differences in regional device sensitivity (four levels: frontal, sensorimotor, temporal, and occipital in the ipsilateral hemisphere). Additionally, a two-way repeated-measures ANOVA was used to compare the different depth-sensitive measurements (three levels: $N_{\text{short}}$, $N_{\text{long}}$, and $V_{\text{long}}$) across time bins (four levels: baseline, 30 to 45 s, 45 to 60 s, and 60 to 75 s). For each time bin and moment, the 5-s average around the maximum change in Δ[HbO], Δ[Hb], $\Delta {\mathrm{StO}}_{2}$, in the ipsilateral hemisphere was analyzed. The $N_{\text{short}}$ was selected to reflect scalp response, $N_{\text{long}}$ provides a signal similar to that of cwNIRS, and $V_{\text{long}}$ is the most sensitive to the brain.

#### 2.5.2 Protocol 2: Phenylephrine administration during shoulder surgery

A one-way ANOVA was used to compare regional differences in device sensitivity (four levels: frontal, sensorimotor, temporal, and occipital) as measured by the percentage of active channels. A Friedman test was used for comparisons if the data were not normally distributed. A paired t-test was run to determine differences in physiological parameters (MAP and HR) before and after PE administration. Additionally, a two-way repeated-measures ANOVA was used to compare the different depth-sensitive measurements (three levels: $N_{\text{short}}$, $N_{\text{long}}$, $V_{\text{long}}$) across time bins (three levels: baseline, 1 to 3.5 min, 3.5 to 6 min). For each time bin and moment, the 5-s average around the maximum change in Δ[HbO], Δ[Hb], and $\Delta {\mathrm{StO}}_{2}$ was analyzed. A one-way ANOVA was also used to assess the regional differences in Δ[HbO] and Δ[Hb], and the time of the minimum desaturation.

## 3 Results

### 3.1 Protocol 1: Digital Carotid Compression

**Fig. 3:**
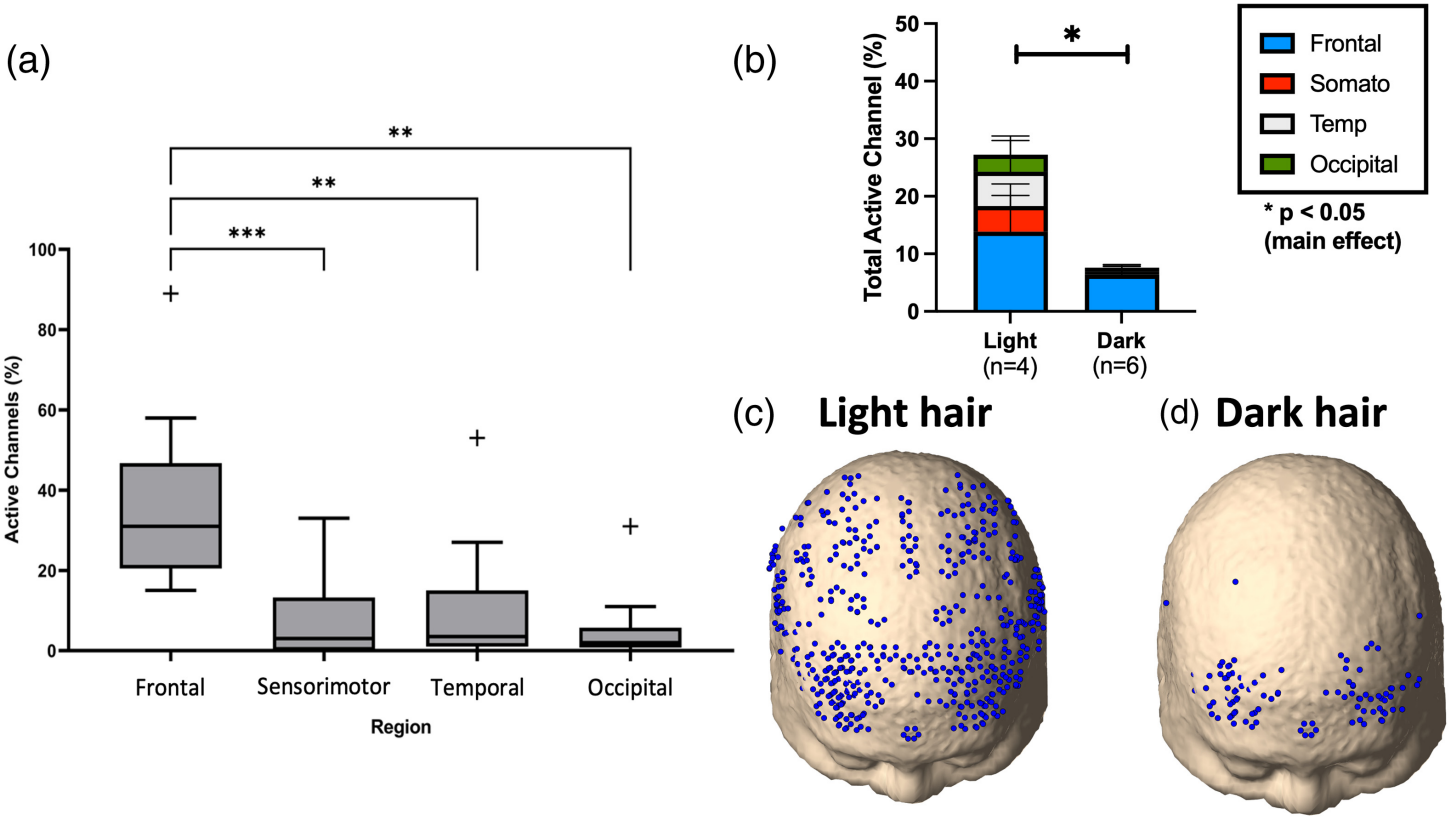
(a) Regional sensitivity of the device (n=10; mean age: 28±5; five females). Box-and-whisker plots show first and third quartiles as outlined by the box, with the middle line indicating the median and the ends of the line indicating minimum and maximum values. Crosses indicate outliers. Asterisks indicate that the frontal region was significantly different from all other regions. (b) “Light” (n=4) and “dark” (n=6) hair participants’ regional sensitivity are displayed as stacked bars of the four regions. An example of the active channel maps overlaid on a standard head model^23^ from a participant from the “light” hair group is shown in panel (c), and an example of the “dark” hair group is shown in panel (d).

Ten subjects were included in the study (28±5 years; five females and five males; Table 1). For the frontal region, 36%±23% of the long-distance channels had good-quality signal, among which 52%±16% were between 25 and 35 mm, 39%±8% between 35 and 45 mm, and 8%±12% between 45 and 60 mm. However, the number or good-quality channels decreased significantly (p≤0.004) for the other regions: 8%±12% for the sensorimotor cortex, 11%±17% for the temporal lobe, and 5%±10% for the occipital lobe [Fig. 3(a)]. Additionally, no significant interaction was found between hair type and region (p=0.3). However, significant main effects of hair type (p≤0.0001) and region (p≤0.0001) were found. Subjects with “light” hair (n=4; three males, one female) had a significantly higher percentage of good-quality channels [27%±25% versus 7%±10%; p≤0.001; Fig. 3(b)] when compared to those with “dark” hair (n=6; two males, four females).

**Fig. 4:**
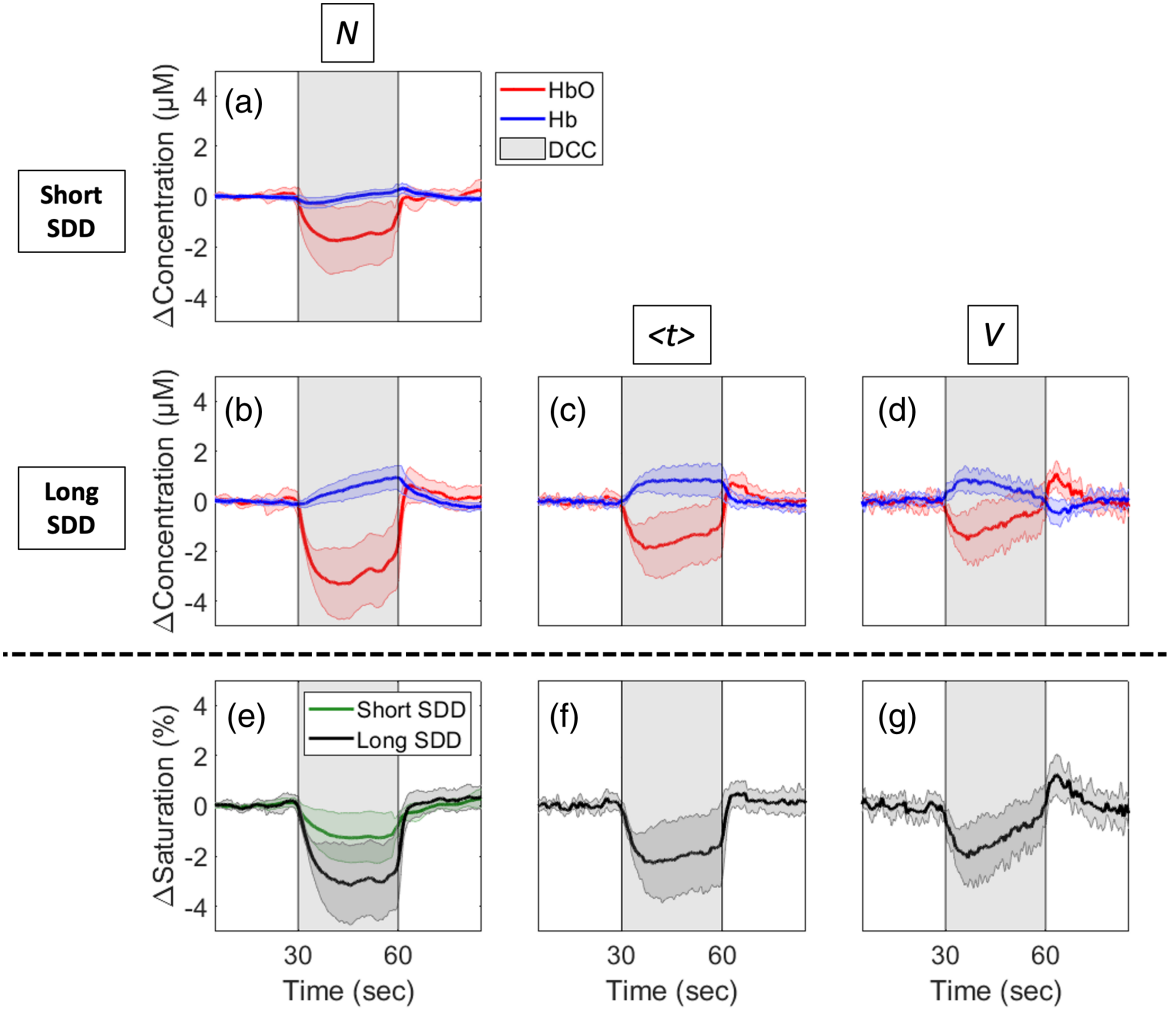
(a)–(d) Average Δ[HbO] (red) and Δ[Hb] (blue) responses to DCC (indicated by the gray-shaded region) in the ipsilateral frontal region. Time courses are presented for the signals measured at short (a) and long (b)–(d) distances. Responses derived from (a) and (b) the total number of photons (N), (c) mean time of flight (⟨t⟩), and (d) variance (V) are shown. (e)–(g) Average long distance (black) and short distance (green) $\Delta {\mathrm{StO}}_{2}$ derived from (e) N, (f) ⟨t⟩, and (g) V are shown. All time courses were averaged across 10 subjects and shading surrounding each line represents the standard deviation.

Given that the frontal was the only region that had good-quality long-distance channels for all the participants [see Figs. 3(c) and 3(d)], it is the only region that could reliably be used to assess differences in the response to DCC across all participants. Figure 4(a) shows Δ[HbO] and Δ[Hb] responses to DCC obtained from the zero-order moment measured at the shortest SDD, $N_{\text{short}}$ (i.e., the measurement that is overwhelmingly sensitive to the extra-cerebral tissues). Figures 4(b)–4(d) display the hemodynamic responses for all three moments extracted from data measured at SDD≥25  mm. The panels are ordered from the least to the most depth-sensitive measurements [moving from Figs. 4(b)–4(d)]. The contralateral time courses are in Fig. S1 in the Supplementary Material but show negligible changes during DCC. Further analysis of the ipsilateral Δ[HbO], Δ[Hb], and $\Delta {\mathrm{StO}}_{2}$ profiles across time revealed a significant interaction in all three parameters across the moments (p≤0.0001 for Δ[HbO], Δ[Hb], and ${\mathrm{StO}}_{2}$)—see Fig. S2 in the Supplementary Material for the complete analysis of significant differences. The most notable differences were the faster recovery during desaturation in the second half of the DCC and the hyperemic response after DCC for $V_{\text{long}}$.

### 3.2 Protocol 2: Phenylephrine Administration During Shoulder Surgery

Data were acquired from 27 shoulder surgery patients. All patients were classified as light-skinned (I or II on the Fitzpatrick skin type scale) and had thinner lighter-colored hair due to age and demographics. Wearing the Kernel flow helmet resulted in a minor skin abrasion for one patient, but no complications were reported for the other subjects. The average age was 65±15 years (14 females and 13 males). Seventeen patients had right shoulder surgery, and ten underwent left shoulder surgery. Only 10 patients received at least one bolus injection of PE, and of these, 5 were excluded due to technical challenges, such as motion artifacts or corrupt files. For consistency, only the first PE injection from each patient was analyzed and compared. Table 2 provides the demographics of the five patients (68±5; two females, two males) included in the analysis and the number of PE injections they received.

To assess the device’s regional sensitivity, all 27 patients were considered. Four patients were excluded from this analysis due to technical issues where some modules could not be connected. For protocol 2, the signal was generally high as the subjects were older and had lower hair density. Overall, the frontal region had 67%±37% of the long-distance channels classified as good quality, the sensorimotor had 71%±32%, the temporal had 59%±35%, and the occipital had 62%±32%. No significant differences were found (p=0.5) in the percentage of good-quality channels between brain regions in this patient population. Resultantly, limiting long distance channels to those with SDD of 25 to 35 mm was suitable for the rest of the analyses.

**Fig. 5:**
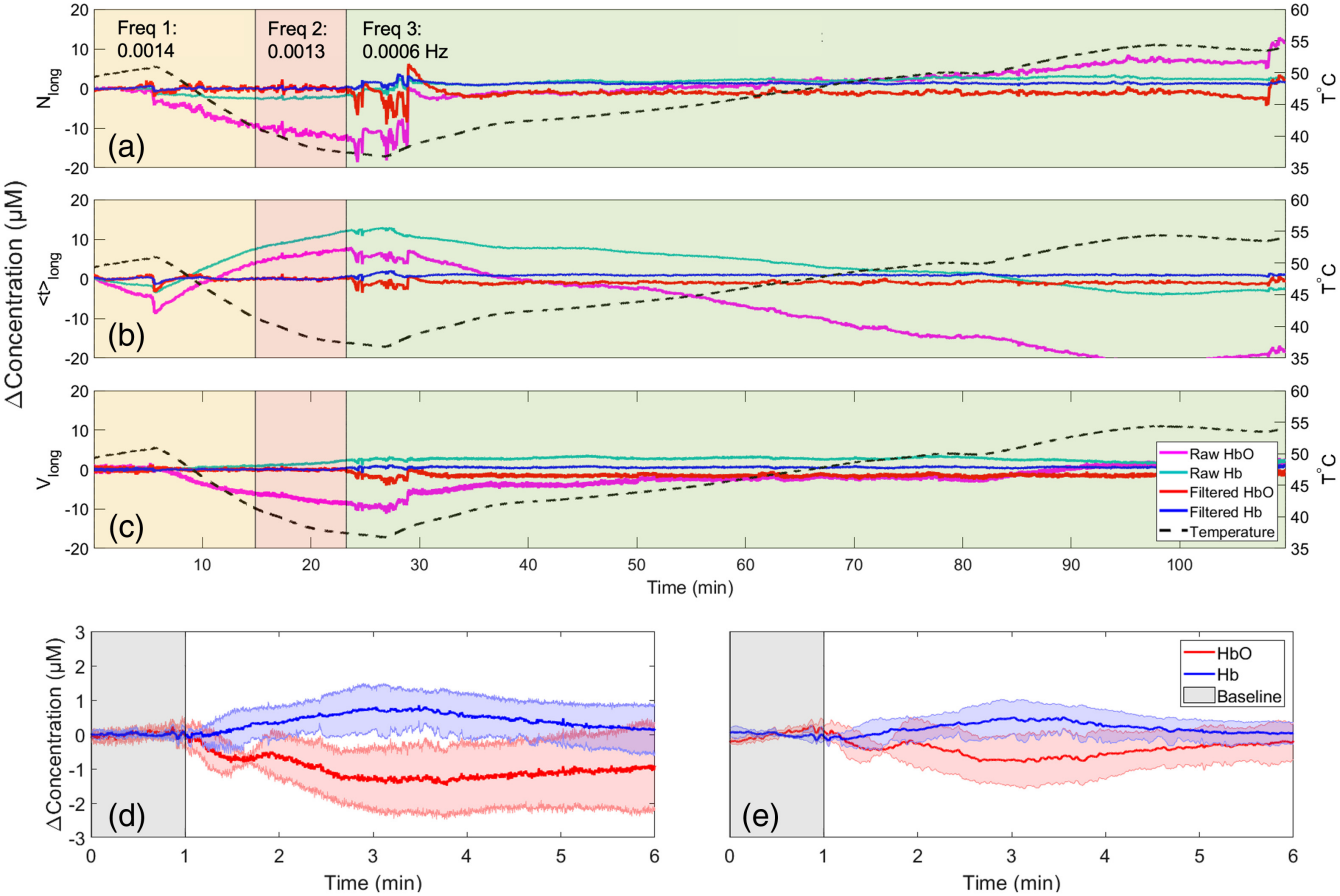
Example time courses of (a) $N_{\text{long}}$, (b) ${\left\langle t\right\rangle }_{\text{long}}$, and (c) $V_{\text{long}}$ from one module over the frontal region of a patient. The overlaid colored boxes indicate windows with different wavelet filter parameters that varied with the corresponding temperature time course (dashed black). For each moment, both the raw and filtered time courses are shown for [HbO] (magenta and red, respectively) and [Hb] (cyan and blue, respectively). Average [HbO] and [Hb] time courses in response to a phenylephrine injection are shown (d) before (e) and after temperature correction. These time courses were calculated from $V_{\text{long}}$. The baseline is displayed in gray, and the response to the PE injection is in white.

An example of temperature-drift correction for each of the three moments is shown in Figs. 5(a)–5(c). In this case, initial $N_{\text{long}}$ and $V_{\text{long}}$ time courses followed the same trend as temperature, whereas ${\left\langle t\right\rangle }_{\text{long}}$ showed the opposite trend. Figures 5(d) and 5(e) present the average Δ[HbO] and Δ[Hb] responses to phenylephrine injection before and after temperature-drift correction. The results demonstrated that temperature correction reduced the variability across subjects, as indicated by the standard deviation, and, more importantly, resulted in the Δ[HbO] and Δ[Hb] time courses returning to baseline.

**Fig. 6:**
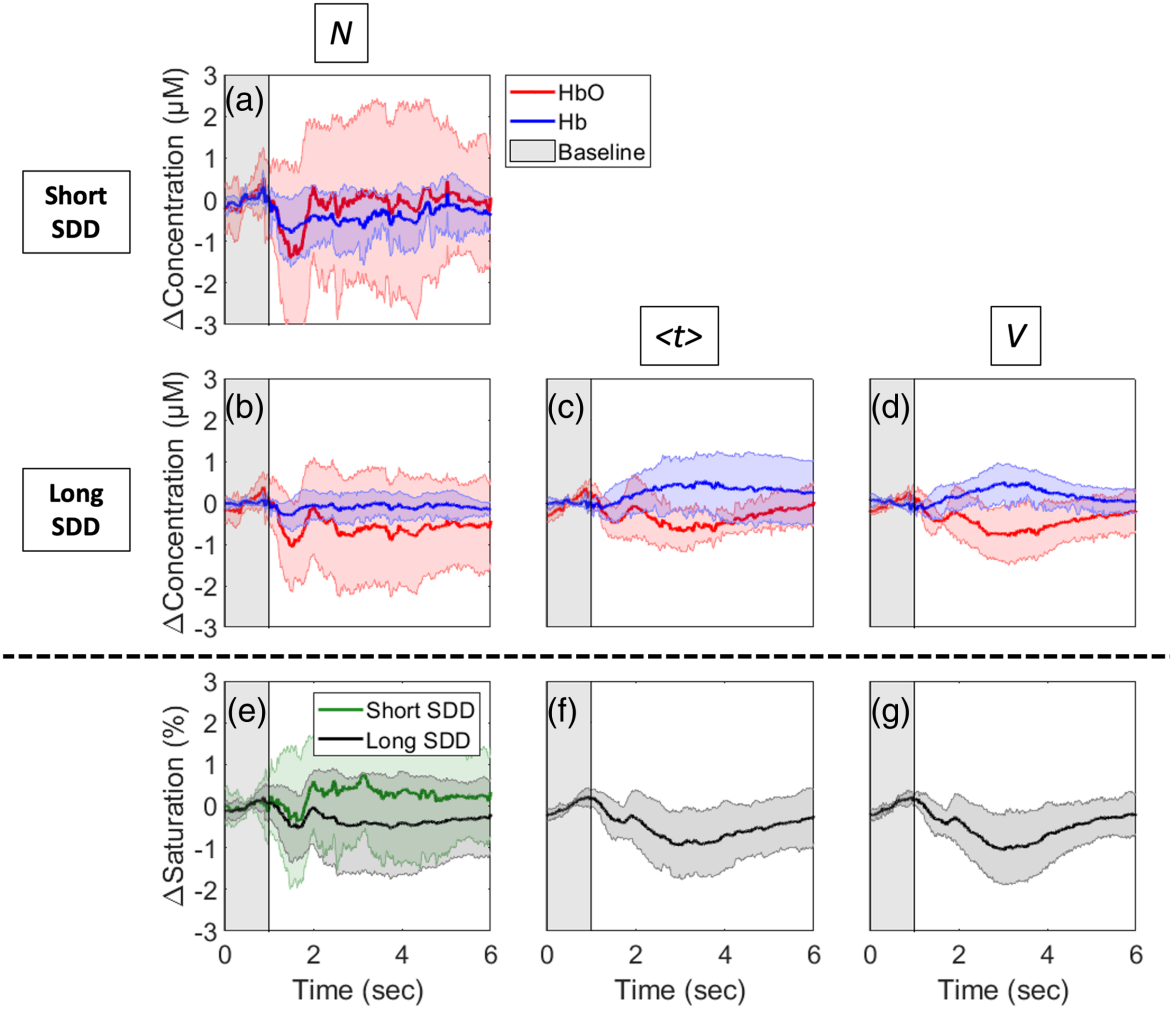
(a)–(d) Average Δ[HbO] (red) and Δ[Hb] (blue) responses to the first phenylephrine bolus injection (indicated by the white region; the baseline is shown in gray) in the left frontal region. Time courses are presented for the signals measured at short (a) and long (b)–(d) Responses derived from (a) and (b) the total number of photons (N), (c) mean time of flight (⟨t⟩), and (d) variance (V) are shown. Average long distance (black) and short distance (green) $\Delta {\mathrm{StO}}_{2}$ derived from (e) N, (f) ⟨t⟩, and (g) V are shown. All time courses were averaged across five subjects and shading surrounding each line represents the standard deviation.

Figure 6 shows the average time courses for the frontal region for patients included in the PE analysis (n=5). The PE injection occurred at the 1-min time point. Through qualitative observation, Fig. 6(a), predominantly the scalp response, showed large variability in the data; however, there appears to be a sharp decrease in Δ[HbO] and Δ[Hb] following PE injection. Qualitatively, in the more brain-sensitive measurements [$V_{\text{long}}$, Fig. 6(d)], there is a pronounced desaturation with a simultaneous decrease in Δ[HbO] and Δ[Hb] increase. Further, the analysis of the time courses of Δ[HbO], Δ[Hb], and $\Delta {\mathrm{StO}}_{2}$ (see Fig. S3 in the Supplementary Material) revealed a significant main effect of time in Δ[HbO] only (p≤0.04) as the baseline was significantly different from the injection period.

Moreover, MAP increased post-injection (61±10  mmHg to 80±12  mmHg; p≤0.01) versus pre-injection, but HR did not significantly change after PE injection (58±10  bpm to 54±11  bpm; p=0.08).

**Fig. 7:**
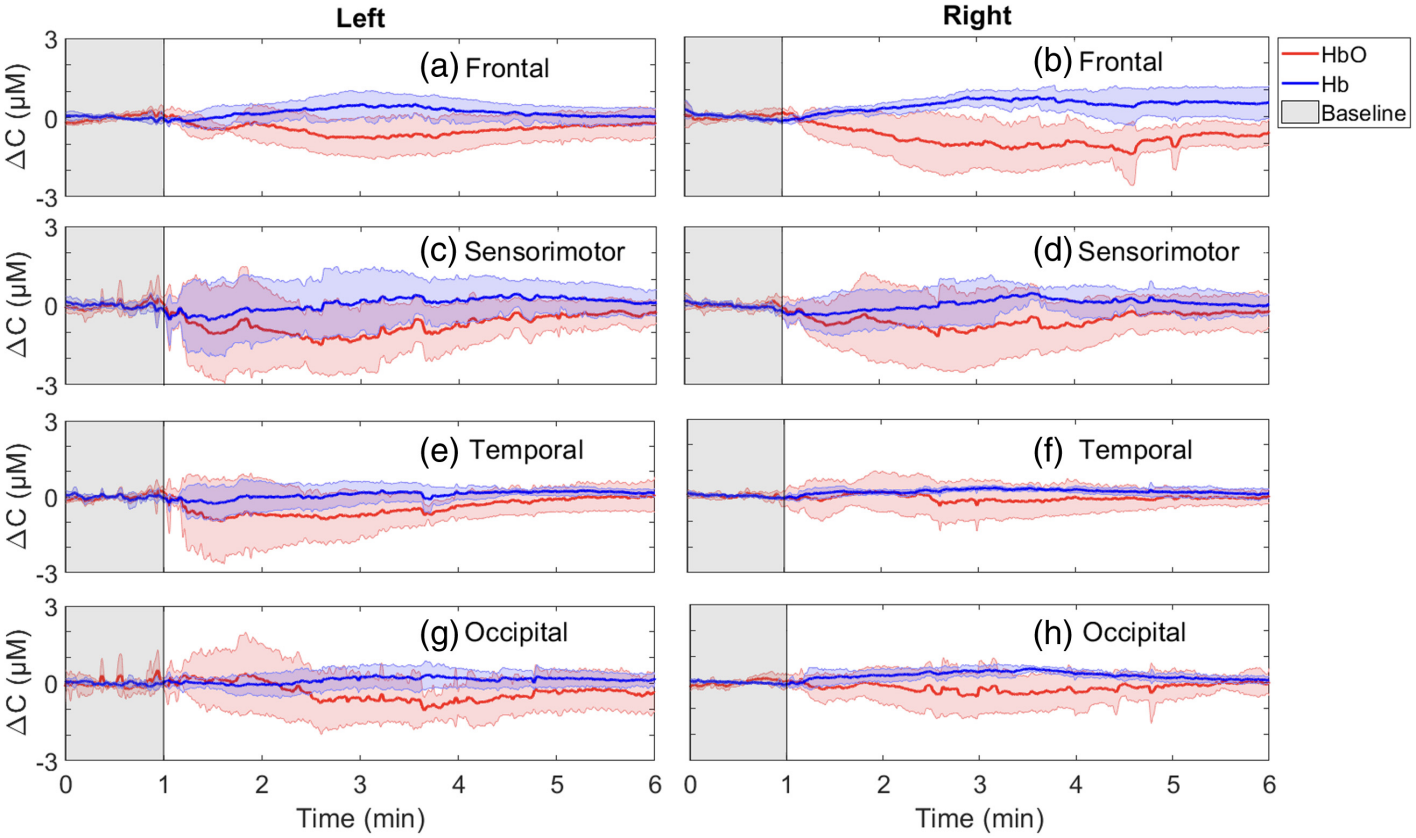
Concentration changes (ΔC) of HbO (red), and Hb (blue), calculated using $V_{\text{long}}$, showing the average (n=5) first response to phenylephrine bolus injection during surgery across the four brain regions (frontal, sensorimotor, temporal, and occipital). The shading indicates the standard deviation across participants.

Figure 7 displays the average time courses (n=5) in all brain regions, calculated using $V_{\text{long}}$. The curves show that all regions had a similar response to the PE injection, characterized by a simultaneous decrease in Δ[HbO] and increase in Δ[Hb]. There were no significant differences in the regional responses to PE injection for the magnitude of Δ[HbO] (p=0.7) or Δ[Hb] (p=0.7) or time of maximum change (p=0.99).

## 4 Discussion

This work investigated the feasibility of using a novel high-density full-head coverage time-resolved system for regional cerebral oxygenation monitoring. Two protocols were conducted: (1) DCCs in healthy volunteers and (2) PE administration during shoulder surgery. The two objectives were to investigate the system’s ability to measure oxygenation simultaneously across all brain regions and compare cerebral hemodynamic responses computed using time-resolved parameters with different depth sensitivities.

### 4.1 Protocol 1: Digital Carotid Compression

In the DCC protocol, the device’s sensitivity was assessed in young healthy subjects by calculating the percentage of good-quality channels across the head. The analysis revealed that the frontal region had a significantly higher number of good-quality channels compared to the sensorimotor, temporal, and occipital regions. Additionally, the device had better sensitivity in subjects with light hair than those with dark hair. These findings indicate that signal quality depends on the type and density of hair. Consequently, using the device for full-head neuromonitoring in patients with darker and longer hair would be challenging. Further, the regional distribution of channel sensitivity reported in this work showed that the frontal region had the most signal while the occipital region had the lowest percentage of good-quality channels. This is consistent with the findings of Ortega-Martinez et al.,^32^ who reported a similar sensitivity distribution with the Kernel Flow. Due to the lower overall signals in the younger participants recruited for protocol 1, a large range of SDD values was used in the analysis. The similarity in the time courses for SDDs 25 to 35 mm and those 35 to 45 mm (see Fig. S4 in the Supplementary Material) indicates that this approach was reasonable. Ideally, improving the signal-to-noise ratio would enable a narrower range of SDD to be selected.

Since the frontal region (measured through the forehead) consistently had the best signal in all participants, this region was used to assess the device’s depth sensitivity in the DCC protocol. Additionally, only ipsilateral hemisphere time courses were analyzed, as the contralateral measurements showed negligible changes during DCC, as expected. The hemodynamic responses to the DCC challenge computed using the various depth-sensitive measurements differed significantly. The measurement known to have the most sensitivity to the brain ($V_{\text{long}}$) showed a faster recovery from desaturation in the second half of the DCC, which may be attributed to collateral flow in the brain that is not possible in the scalp. The larger decrease in saturation observed in $N_{\text{long}}$ compared to $N_{\text{short}}$ agrees with our previous work^18^ using a cwNIRS system, which found a larger reduction in saturation in the long SDDs compared to the short SDDs in response to DCC. The long-distance cwNIRS measurements were previously attributed to the brain; however, the smaller decrease in saturation in $V_{\text{long}}$ observed in this study suggests that cerebral desaturation following PE administration is likely smaller than previously suggested.^18^ Additionally, Δ[Hb] increase was only visible in the long-distance measurements, which indicates greater oxygen extraction in the brain during DCC compared to the scalp, reflecting the lack of oxygen stores and higher energy metabolism in the brain.

Finally, a cerebral ($V_{\text{long}}$) hyperemic response was observed during the recovery in the Δ[HbO] and $\Delta {\mathrm{StO}}_{2}$ as blood flow increased following occlusion release. Similar hyperemia has been reported in our previous work using diffuse correlation spectroscopy^18^ but was not evident in the ${\mathrm{StO}}_{2}$ values using cwNIRS. This may be due to the additional depth-sensitivity of $V_{\text{long}}$, as the hyperemia is also not evident in the $N_{\text{long}}$ in the current study. Diffuse correlation spectroscopy provides inherently better depth-sensitivity than cwNIRS due to the substantially higher blood flow in the brain compared to the scalp,^33^^,^^34^ which can explain this difference. Additionally, these findings agree with Sorteberg et al. who performed DCC on patients with neck neoplasms or traumatic carotid-cavernous fistulas,^17^ and found that the blood velocity in the middle cerebral artery (measured using transcranial Doppler ultrasound) showed a hyperemic response following DCC.

### 4.2 Protocol 2: Phenylephrine Administration During Shoulder Surgery

There were no significant differences in device sensitivity between brain regions in the surgery data. This finding contrasts with the first protocol in which the frontal region had the best signal. This difference is likely due to the difference in demographics between the two studies. For the surgery study, subjects were older, had lighter-colored hair, and reduced hair density. Therefore, these results agree with the findings of the first protocol that those with “light” hair had better overall signal. The better sensitivity of the device in surgery patients is promising since the aim was to assess the device intraoperatively.^35^

An unexpected challenge with neuromonitoring over longer periods with the Kernel flow was overheating. An air-cooling device was used to blow cool air on the device; however, there were still temperature-induced signal drifts (see Fig. 5). A dynamic wavelet bandpass filtering approach to remove very low frequencies (below 0.008 Hz or 125 s) was implemented to correct for these signal drifts and very high frequencies (above 3.1 Hz) for motion artifacts. This wavelet filtering proved crucial, as evident by comparing $N_{\text{long}}$, ${\left\langle t\right\rangle }_{\text{long}}$, and $V_{\text{long}}$ time series before and after applying temperature correction (Fig. 5). It is possible that wavelet filtering may have removed physiological signals outside the bandpass filter. However, this study focused on the rapid cerebral hemodynamic response to a vasopressor, which occurred over a much shorter time frame than those caused by temperature instability.

After 5 min of PE injection, the MAP settles into a new elevated baseline; therefore, it is possible that the hemodynamic parameters settle into a new baseline as well. In this study, however, the filtered Δ[HbO] and Δ[Hb] returned to the pre-PE values, and not a new baseline [see Fig. 5(e)]. This finding agrees with the work of Meng et al.^36^ and Fassaert et al.^37^ who likewise demonstrated that while cerebral oxygenation returned to baseline within 5 min, the recovery of basal MAP typically took longer, likely reflecting differences in cerebral and systemic vascular regulation.

The injection of PE caused the expected systemic response, i.e., an increase in MAP consistent with other studies.^38^^,^^39^ The slight decrease in HR (although not significant) was also expected and has been reported in the literature.^39^ In terms of the NIRS measurements, $N_{\text{short}}$, which is assumed to predominantly reflect scalp hemodynamics, showed a sharp decrease in Δ[HbO], potentially driven by the vasoconstrictive effects of PE on alpha receptors present in scalp tissue. However, a larger sample size would help investigate this further as the time courses had large variability. Desaturation events, characterized by a simultaneous increase in Δ[Hb] and decrease in Δ[HbO], were observed in the $V_{\text{long}}$, the most brain-sensitive measurement. The cerebral desaturation agrees with previous studies using cwNIRS devices, which report decreases as large as 14% in cerebral oxygen saturation^40^; however, the desaturation reported in the current work are smaller (less than 2%) and likely not clinically significant. Previous studies have shown that the magnitude of ${\mathrm{StO}}_{2}$ changes in response to PE decreases with increased brain sensitivity.^20^^,^^40^^,^^41^ Additionally, Rosenthal et al.^42^ detected very small changes in cerebral blood flow in response to phenylephrine administration, which is consistent with the small changes in the time-resolved measurements in this work. Our findings also agree with other studies using more depth-sensitive NIRS techniques, such as frequency-domain NIRS (fdNIRS).^21^ It has been suggested that the previously reported cerebral desaturation following PE injection may be related to signal contamination due to extracranial vasoconstriction.^43^ Importantly, recent work utilizing direct measurements of cerebral tissue oxygen tension in open rat brains^44^ suggests that PE injection does not result in cerebral desaturation. Therefore, despite the well-known superior depth sensitivity of trNIRS,^15^ the small magnitude of desaturation observed in $V_{\text{long}}$ may be due to residual scalp contamination. Moreover, the small decreases in Δ[HbO] and increases in Δ[Hb] may be due to changes in cerebral artery vasomotor tone. That is, a small drop in arterial blood volume due to vasoconstriction could shift the measurements to the venous side, leading to the observed trends.

There were no statistically significant differences in the regional response to PE. It is important to note that although there were no significant differences, this may be due to the smaller sample size (only five patients received PE). A subsequent larger study could provide more conclusive answers to potential regional differences in response to PE administration.

### 4.3 Limitations

There were a few limitations to this work. First, access to the data in the rawest form (before the automatic processing through the Kernel Flow pipeline) would be helpful to assess why channels were discarded within the automated pipeline and allow us to evaluate the stability and quality of the recorded DTOFs. Additionally, it remains unclear if potential regional differences in hemodynamic responses result from actual physiological differences. They could result from sensitivity differences across the head due to the data processing that uses sensitivity factors to estimate the hemodynamic response. Using magnetic resonance imaging, Wu et al. found that the skull thickness varies across the head;^45^ however, this was not accounted for in the sensitivity factors and could impact our conclusion on the lack of regional differences.

### 4.4 Future Work

Overcoming the current hair limitation would help detect potential regional differences in the cerebral response to DCC when comparing regions supplied by different cerebral vasculatures. Due to the limited variability in skin tone in the current study, the analysis focused on hair and not skin tone. A larger and more diverse sample group would allow an analysis of the impact of both hair and skin types on device sensitivity. It would also be useful to conduct studies using tissue-mimicking phantoms to better understand the temporal stability of the Kernel Flow. A further consideration is that most surgeries are performed with the patient supine, which would require testing to ensure the device provides sufficient contact across the head when in this position.

## 5 Conclusions

In summary, this study demonstrated the feasibility and potential of using a high-density, full-head coverage trNIRS device for adult neuromonitoring using two study protocols: DCC and PE administration during shoulder surgery. The device’s sensitivity depended on the presence and type of hair, particularly in the first protocol, which involved young volunteers. The second protocol showed improved signal when using the device on shoulder surgery patients who were older and had decreased hair density. Notably, the age of candidates for neurovascular surgery is typically higher than those recruited for the first protocol, which may alleviate this limitation. Analysis of the device’s depth-sensitivity showed distinct scalp and brain responses, which shows promise. The use of the device for intraoperative neuromonitoring demonstrated the feasibility of its clinical translation. A larger-scale study would shed more light on potential regional differences due to DCC and PE administration.

## Supplementary Material



## Data Availability

Data can be made available by contacting the authors. Because of the participant consent obtained as part of the recruitment process, making these data publicly available is not possible.
