# Supplementary material for: Assessing changes in regional cerebral hemodynamics in adults with a high-density full-head coverage time-resolved near-infrared spectroscopy device
Source: J Biomed Opt. 2024 May 3;29(Suppl 3):S33302. doi: 10.1117/1.JBO.29.S3.S33302 (PMC11068267; doi:10.1117/1.JBO.29.S3.S33302)
Supplement: Supplementary file 1 [file JBO_029_S33302_SD001.pdf]

Supplementary Material

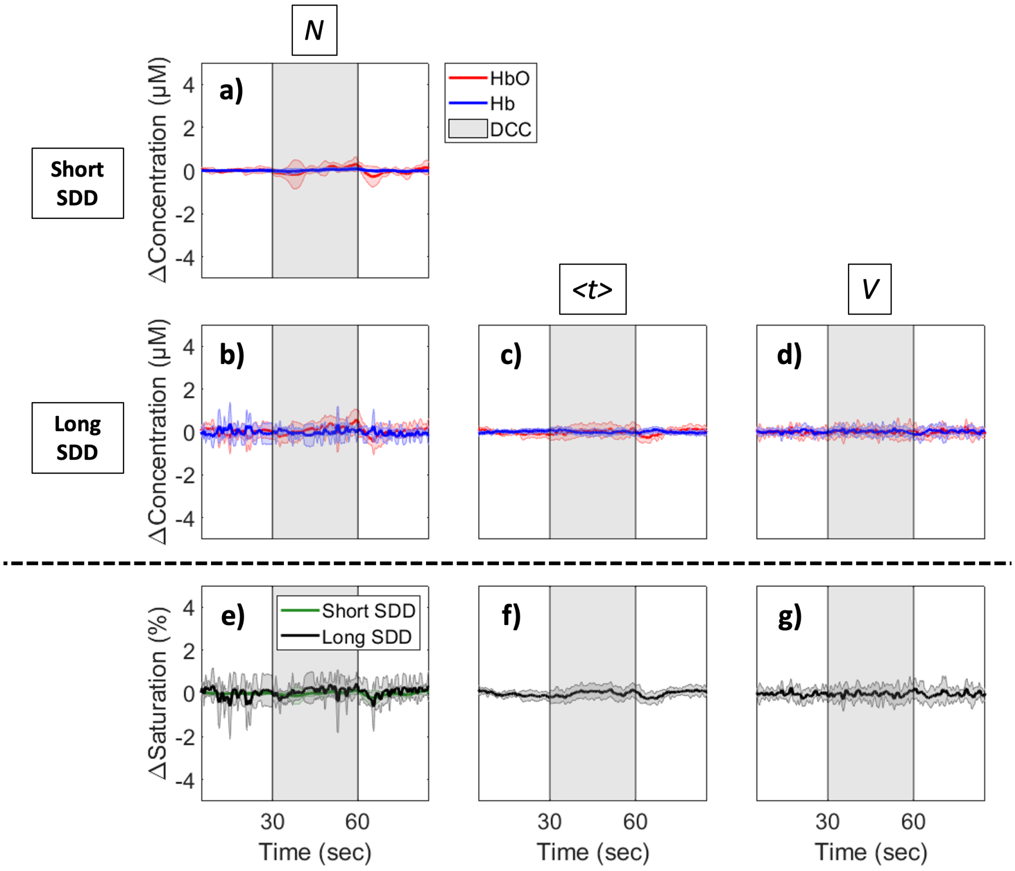

Figure S1 (a)-(d) Average $\Delta[\mathbf{HbO}]$ (red) and $\Delta[\mathbf{Hb}]$ (blue) responses to digital carotid compression (DCC) (indicated by the grey-shaded region) in the contralateral frontal region. Time courses are presented for the signals measured at short (a) and long (b-d) distances. Responses derived from (a-b) the total number of photons (*N*), (c) mean time of flight (*<t>),* and (d) variance (*V*) are shown. (e)-(g) Average long distance $\mathbf{\Delta StO_2}$ (black) and short distance (green) derived from (e) *N*, (f) *<t>,* and (g) *V* are shown. All time courses were averaged across ten subjects, and shading surrounding each line represents the standard deviation.

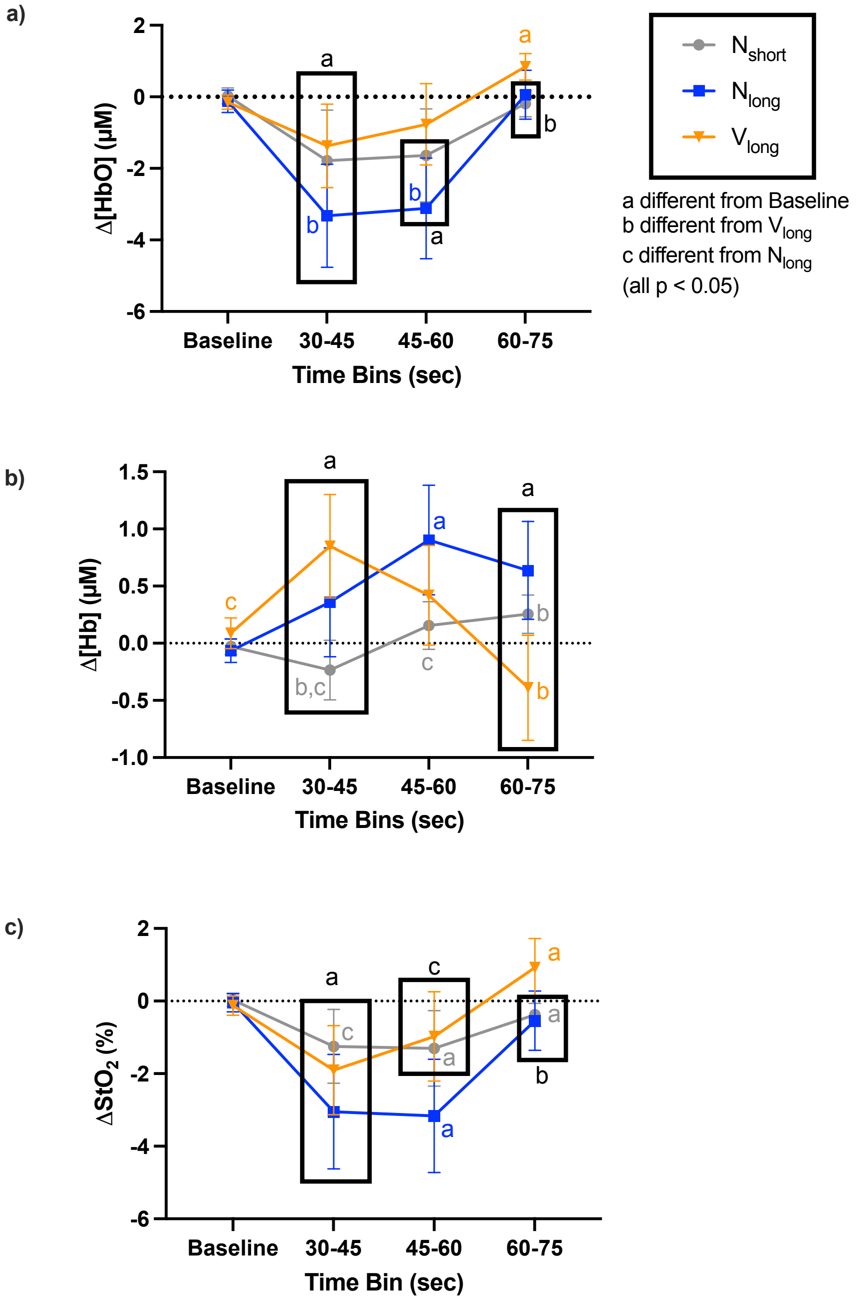

Figure S2 Full statistical analysis for Protocol 1. Two-way repeated-measures ANOVA across the different depth-sensitive measurements (3 levels: N_{short} (gray), N_{long} (blue), V_{long} (orange)) and across time bins (4 levels: baseline, 30-45 s, 45-60 s, 60-75 s). For each time bin and moment, the 5-second average around the maximal change in (a) Δ[HbO], (b) Δ[Hb], (c) ΔStO_2, in the ipsilateral hemisphere (Fig. 4). A significant interaction was seen in all 3 parameters. Dunnet post-hoc analysis showed refined comparisons.

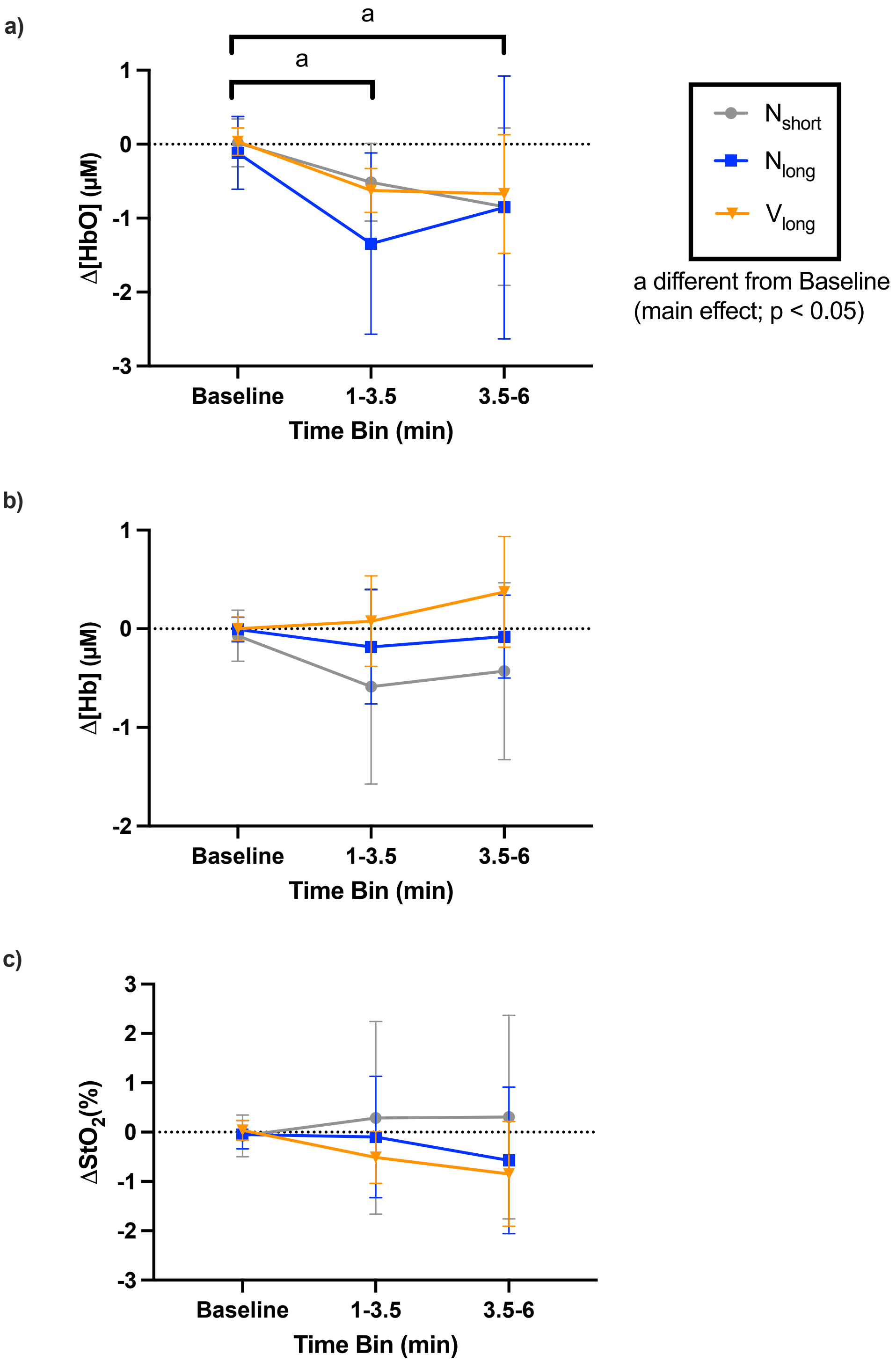

Figure S3 Full statistical analysis for Protocol 2. Two-way repeated-measures ANOVA across the different depth-sensitive measurements (3 levels: N_{short} (gray), N_{long} (blue), V_{long} (orange)) and across time bins (3 levels: baseline, 1-3.5 min, 3.5-6 min). For each time bin and moment, the 5-second average around the maximal change in (a) Δ[HbO], (b) Δ[Hb], and (c) ΔStO2 was analyzed (Fig. 6). Only Δ[HbO] showed a significant main effect of time.

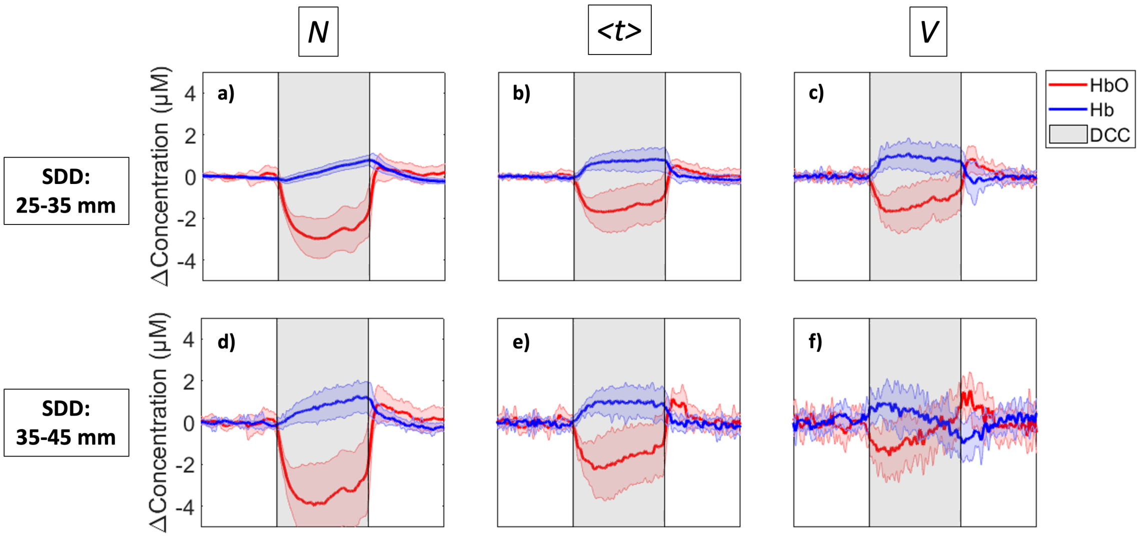

Figure S4 (a)-(f) Average Δ[**HbO**] (red) and Δ[**Hb**] (blue) responses to digital carotid compression (DCC) (indicated by the grey-shaded region) in the ipsilateral frontal region. Time courses are presented for the signals measured at (a-c) SDD between 25-35 mm and at (d-f) SDD between 35-45 mm. Responses derived from (a,d) the total number of photons (*N*), (b,e) mean time of flight (<*t*>), and (c,f) variance (*V*) are shown.
